# A novel GH10 xylanase from *Penicillium* sp. accelerates saccharification of alkaline-pretreated bagasse by an enzyme from recombinant *Trichoderma reesei* expressing *Aspergillus* β-glucosidase

**DOI:** 10.1186/s13068-017-0970-2

**Published:** 2017-11-21

**Authors:** Nozomu Shibata, Mari Suetsugu, Hiroshi Kakeshita, Kazuaki Igarashi, Hiroshi Hagihara, Yasushi Takimura

**Affiliations:** Biological Science Research, Kao Corporation, 1334 Minato, Wakayama, Wakayama 640-8580 Japan

**Keywords:** *Trichoderma reesei*, *Penicillium* sp., Cellulase, GH10 xylanase, Biomass saccharification, Carbohydrate-binding module family 1, Alkaline-pretreated bagasse

## Abstract

**Background:**

*Trichoderma reesei* is considered a candidate fungal enzyme producer for the economic saccharification of cellulosic biomass. However, performance of the saccharifying enzymes produced by *T. reesei* is insufficient. Therefore, many attempts have been made to improve its performance by heterologous protein expression. In this study, to increase the conversion efficiency of alkaline-pretreated bagasse to sugars, we conducted screening of biomass-degrading enzymes that showed synergistic effects with enzyme preparations produced by recombinant *T. reesei*.

**Results:**

*Penicillium* sp. strain KSM-F532 produced the most effective enzyme to promote the saccharification of alkaline-pretreated bagasse. Biomass-degrading enzymes from strain KSM-F532 were fractionated and analyzed, and a xylanase, named PspXyn10, was identified. The amino acid sequence of PspXyn10 was determined by cDNA analysis: the enzyme shows a modular structure consisting of glycoside hydrolase family 10 (GH10) and carbohydrate-binding module family 1 (CBM1) domains. Purified PspXyn10 was prepared from the supernatant of a recombinant *T. reesei* strain. The molecular weight of PspXyn10 was estimated to be 55 kDa, and its optimal temperature and pH for xylanase activity were 75 °C and pH 4.5, respectively. More than 80% of the xylanase activity was maintained at 65 °C for 10 min. With beechwood xylan as the substrate, the enzyme had a *K*_m_ of 2.2 mg/mL and a *V*_max_ of 332 μmol/min/mg. PspXyn10ΔCBM, which lacked the CBM1 domain, was prepared by limited proteolysis. PspXyn10ΔCBM showed increased activity against soluble xylan, but decreased saccharification efficiency of alkaline-pretreated bagasse. This result indicated that the CBM1 domain of PspXyn10 contributes to the enhancement of the saccharification efficiency of alkaline-pretreated bagasse. A recombinant *T. reesei* strain, named X2PX10, was constructed from strain X3AB1. X3AB1 is an *Aspergillus aculeatus* β-glucosidase-expressing *T. reesei* PC-3-7. X2PX10 also expressed PspXyn10 under the control of the *xyn2* promoter. An enzyme preparation from X2PX10 showed almost the same saccharification efficiency of alkaline-pretreated bagasse at half the enzyme dosage as that used for an enzyme preparation from X3AB1.

**Conclusions:**

Our results suggest that PspXyn10 promotes the saccharification of alkaline-pretreated bagasse more efficiently than TrXyn3, a GH10 family xylanase from *T. reesei*, and that the PspXyn10-expressing strain is suitable for enzyme production for biomass saccharification.

**Electronic supplementary material:**

The online version of this article (10.1186/s13068-017-0970-2) contains supplementary material, which is available to authorized users.

## Background

Cellulose and hemicellulose, naturally produced by plants as cell wall components, are the two most abundant polysaccharides on Earth. To help address environmental problems, attempts have been made to produce saccharides from these polysaccharides, and to convert them to chemicals such as bioethanol by fermentation [1]. These polysaccharides can be converted to fermentable sugars by saccharifying enzymes such as cellulase and hemicellulase. Filamentous fungi, including species of *Trichoderma*, *Penicillium*, and *Aspergillus*, produce these saccharifying enzymes [2].

Cellulases are classified into three major types: cellobiohydrolases (CBH, EC 3.2.1.91) endo-β-1,4-glucanases (EG, EC 3.2.1.4), and β-glucosidases (BGL, EC 3.2.1.21). CBH releases cellobiose from the ends of crystalline cellulose. EG cleaves internal bonds of amorphous cellulose. BGL converts cellobiose and cello-oligosaccharides into glucose. Recently, lytic polysaccharide monooxygenase (LMPO, formerly glycoside hydrolase family 61) has also been shown to degrade cellulose, by cleavage of cellulose chains with oxidation [3].

Hemicellulases are classified as endo-β-1,4-xylanases (XYN, EC 3.2.1.8), β-xylosidases (BXL, EC 3.2.1.37), or other accessory enzymes. XYN cleaves the internal bonds of xylan, which is the main chain of hemicellulose. BXL releases xylose from xylo-oligosaccharides. Accessory enzymes, including α-*N*-arabinofuranosidase (ABF, EC 3.2.1.55), feruloyl esterase (FAE, EC 3.1.1.73), and acetyl xylan esterase (AXE, EC 3.1.1.72), cleave the side-chains of hemicellulose.

Enzymatic hydrolysis of cellulosic biomass requires a high dose of cellulase because of the complex structure of crystalline cellulose as it is surrounded by hemicellulose and lignin, and the fact that cellulases suffer from end product inhibition [4]. For economic saccharification of cellulosic biomass, reducing the cost of both the raw materials and enzymes and improvement of enzymatic saccharification performance are required [5].

To reduce the cost of cellulase enzymes, many studies have focused on cellulase production by filamentous fungi, such as *Aspergillus, Penicillium,* and *Trichoderma*, which are known to produce substantial amounts of cellulases [6–8]. Among them, *Trichoderma reesei,* an anamorph of *Hypocrea jecorina* and one of the best-studied cellulolytic fungi, produces a complete set of cellulases that can degrade cellulose to glucose [9]. In addition, its protein productivity has made this fungus an important commercial enzyme producer. Because of the industrial usefulness of *T. reesei,* many studies on improving its cellulase productivity by random mutagenesis and genetic modification have been reported. The hyper-secreting mutant *T. reesei* Rut-C30, which is one of the most widely used strains for production of cellulolytic enzymes, was obtained after three rounds of mutagenesis of the wild-type strain QM6a [10]. This strain is capable of cellulase and hemicellulase production on glucose-containing media because of mutations in *cre1* (encoding the carbon catabolite repressor protein) [11]. In Japan, *T. reesei* strain PC-3-7 was developed following several rounds of random mutagenesis from *T. reesei* QM9414, a mutant with enhanced cellulase activity derived from QM6a [12–15]. The PC-3-7 strain showed twice as much cellulase activity as QM9414 when induced by α-sophorose, because of mutations in *cre1* and *bgl2* (encoding intracellular β-glucosidase) [15, 16]. Moreover, Denton et al. have reported that disruption of *cre2*, a gene encoding a deubiquitinating enzyme, in *T. reesei* led to increase a threefold increase in cellulase activity when induced by microcrystalline cellulose [17], and Zhang et al. have reported that disruption of the alkaline serine protease in *T. reesei* resulted in enhancement of heterologous protein productivity [18]. As a result of such developments, it has been reported that *Trichoderma* mutants produce enzymes up to 100 g/L in controlled fermenter cultivations [19], thus making it possible to greatly reduce the cost of saccharifying enzymes.

To achieve the economic saccharification of cellulosic biomass, both the cost and the performance of the enzyme must be optimized. Attempts have been made to increase the performance of the saccharifying enzyme produced by *T. reesei*. It was reported that although the enzyme produced by *T. reesei* contains high activities of both CBH and EG, it contains quite low BGL activity [20]. The low BGL activity causes cellobiose accumulation and subsequent product inhibition of CBH1 (Cel7a), resulting in reduced biomass conversion efficiency [21, 22]. To overcome this problem, homologous and heterologous expressions of BGL have been investigated [23, 24]. Zhang et al. have reported that enzyme from recombinant *T. reesei* expressing BGL1 of *T. reesei* (TrBGL1) shows enhanced filter paper activity, and Wang et al. have reported heterologous expression of BGL1 from *Aspergillus niger* (AnBGL1) in *T. reesei*. Characterization of BGL1 from *A. aculeatus* (AaBGL1) was also reported [25, 26], and it was found that this enzyme showed higher specific activity against cellobiose than TrBGL1 (AaBGL1 has an activity of 180 U/mg, while that of TrBGL1 is 19 U/mg) [27], and a higher Ki value for glucose than AnBGL1 (AaBGL1 has a Ki of 10 mM, while that of AnBGL1 is 3 mM) [26, 28]. Therefore, Nakazawa et al. constructed three AaBGL1-expressing *T. reesei* strains, X3AB1, C1AB1, and E1AB1 to enhance the performance of cellulase [27, 29]. Strains X3AB1, C1AB1, and E1AB1 were derivatives of *T. reesei* strain PC-3-7 containing an *aabg1* cDNA under the control of the *xyn3*, *cbh1*, and *eg1* promoters, respectively. X3AB1 was homologously recombined at the *xyn3* locus, and therefore this strain did not harbor the *xyn3* gene in the genome. Enzyme preparations named JN11H, JN12H, and JN13H were, respectively, produced from strains X3AB1, C1AB1, and E1AB1 grown on cellulose and xylan as a carbon source, and JN11H and JN13H showed almost the same saccharification efficiency against various substrates at one-fifth or less dosage of an enzyme preparation from PC-3-7 [29, 30]. As a result of such studies, the dosage of enzymes for saccharification has been reduced. However, the proportion of the enzyme cost to the production cost of sugar and bioethanol is still high [31]. When aiming for more economic sugar production, not only further improvement of the enzyme performance but also adjustment of the enzyme to an inexpensive substrate is important.

For economic cellulosic ethanol production, it is economically advantageous to use waste and byproduct biomass. One of these byproducts is sugarcane bagasse. Bagasse is a residue obtained from sugarcane after it is crushed to obtain the juice used for sugar production. It was reported that ~ 279 million metric tons of sugarcane residues (sugarcane bagasse and leaves) are generated every year in the world and about half of sugarcane bagasse remains unused [32, 33]. Therefore, the bioconversion of leftover bagasse may lead to more economic bioethanol production.

It was of interest to develop an enzyme preparation suitable for saccharification of bagasse subjected to the commonly used method of alkaline pretreatment [34]. In this study, we conducted screening of biomass-degrading enzymes that showed synergistic effects with enzyme preparations produced by recombinant *T. reesei* expressing *Aspergillus* BGL. A novel glycoside hydrolase family 10 (GH10) xylanase was obtained from *Penicillium* sp. KSM-F532. Then, a *Penicillium* xylanase-expressing *T. reesei* was constructed, and the enzyme preparation from this strain was compared with JN11H and JN13H.

## Methods

### Strains and materials

Strains are listed in Table 1. *T. reesei* PC-3-7 (ATCC 66589) was obtained from Kyowa Hakko Bio Co., Ltd. (Tokyo, Japan). *T, reesei strains* X3AB1, X3AB1Δ*pyr4* (uracil auxotroph), and E1AB1 were kindly provided by Prof. W. Ogasawara (Nagaoka University of Technology). Strains were maintained on potato dextrose agar (PDA, Difco Laboratories, Detroit, USA) plates.

**Table 1:** *Trichoderma reesei* strains and derived enzyme preparations

Strains	Genotypes	Enzyme preparations^a^
PC-3-7	Wild-type	
X3AB1	*amdS* ^+^, *xyn3*p::*aabg1*, Δ*xyn3*::*aabg1*	JN11H
E1AB1	*amdS* ^+^, *eg1*p::*aabg1*	JN13H
X3AB1Δ*pyr4*	*amdS* ^+^, *xyn3*p::*aabg1*, Δ*xyn3*::*aabg1,* Δ*pyr4*	
C1PX10	*amdS* ^+^, *cbh1*p::*pspxyn10*, Δ*cbh1*::*pspxyn10*	
X2PX10	*amdS* ^+^, *xyn3*p::*aabg1*, Δ*xyn3*::*aabg1*, *xyn2*p::*pspxyn10*	JNK25H

^a^Indicated strains were grown in media with 10% (w/v) microcrystalline cellulose and 2% (w/v) beechwood xylan

Alkaline-pretreated bagasse was used as the saccharification substrate. A total of 3 kg of sugarcane bagasse, collected from Okinawa, Japan, was treated with 3 L of 1% (w/v; weight per volume) sodium hydroxide solution using 50 L reactor (KMJ-501 MGU, Mitsuwa Frontech Corp., Osaka, Japan) for 20 min at 120 °C. The pretreated bagasse was harvested from the slurry by centrifugation at 536×*g* for 5 min and washed with tap water until the pH was neutral. Two batches of the obtained biomass were resuspended in 30-L of tap water and harvested by centrifugation. The components of the substrate were cellulose, 51 (%w/w); hemicellulose, 28; lignin, 7.8; and ash, 2.2, as determined by high-performance liquid chromatography (HPLC) using a two-step acid hydrolysis according to the Laboratory Analytical Procedure (LAP) [35]. Glucose, xylose, mannose, galactose, arabinose, and cellobiose were analyzed by HPLC (Prominence, Shimadzu, Kyoto, Japan) using an Asahipak NH2P-50 4E 4.6 mm × 250 mm (Shodex, Tokyo, Japan) carbohydrate column [27]. This substrate and the component information were kindly provided by Dr. Y. Kobayashi (Japan Bioindustry Association).

Xyn3 of *T. reesei* (TrXyn3) was kindly provided by Dr. K. Yaoi. TrXyn3 was produced by *Pichia pastoris* and purified as reported [36].

### Enzyme productions

For preculture for enzyme production, 10^7^ spores of each strain were inoculated into 50 mL of basal medium [14] containing 1% (w/v) glucose in a 500-mL Erlenmeyer flask. Spores were counted using a Thoma hemocytometer (Sunlead Glass Corp., Saitama, Japan). The basal medium comprised 0.14% (w/v) (NH_4_)_2_SO_4_, 0.2% (w/v) KH_2_PO_4_, 0.03% (w/v) CaCl_2_·2H_2_O, 0.03% (w/v) MgSO_4_·7H_2_O, 0.1% (w/v) polypeptone, 0.05% (w/v) yeast extract, 0.1% (w/v) Tween 80, and 0.1%(w/v) trace element solution in 50 mM tartrate-Na buffer (pH 4.0). Trace element solution contained 6 mg of H_3_BO_3_, 26 mg of (NH_4_)_6_Mo_7_O_24_·4H_2_O, 100 mg of FeCl_3_·6H_2_O, 40 mg of CuSO_4_·5H_2_O, 8 mg of MnCl_2_·4H_2_O, and 200 mg of ZnCI_2_ in 100 mL of distilled water. Precultures were carried out with shaking at 220 rpm and 28 °C for 2 days. The main cultures (100 mL of basal medium containing 10% (w/v) microcrystalline cellulose and 2% (w/v) beechwood xylan (Tokyo Kasei Kogyo Co., Tokyo, Japan) in a 250-mL jar fermentor Bio Jr. 8 (Biott Co., Ltd., Tokyo, Japan) were inoculated with 10 mL of the precultures. Main culture was carried out at 28 °C, with 0.5 volumes of air per volume of liquid per minute aeration and 3 ppm dissolved oxygen controlled by mechanical agitation. The pH was controlled at 4.5 using ammonia solution (5% w/v). After 4 days’ cultivation, cells were removed from the culture broth by centrifugation at 10,000×*g* for 20 min, and the supernatant was filtered through a 0.20-μm cellulose acetate membrane filter (13CP020AN; Advantec, Toyo Roshi Kaisha Ltd., Tokyo, Japan). The culture supernatants were used as enzyme preparations. JN11H, JN13H, and JNK25H enzyme preparations were produced from *T. reesei* X3AB1, E1AB1, and X2PX10, respectively. Experiments were carried out in triplicates.

### Isolation of fungi producing biomass-degrading enzymes

Soil samples were collected in Tochigi Prefecture, Japan. A little amount of soil was suspended in sterile water and spread on agar plates containing 0.5% (w/v) powdered cellulose (KC-flock W-50, Nippon Paper Industries Co.), 0.2% (w/v) KH_2_PO_4_, 0.03% (w/v) urea, 0.15% (w/v) yeast extract, 0.25% (w/v) Polypepton, 0.03% (w/v) MgSO_4_·7H_2_O, 0.03% (w/v) CaCl_2_·6H_2_O, 0.14% (w/v) (NH_4_)_2_SO_4_, 2.0% (w/v) agar, 0.002% (w/v) Congo red, and 0.01% (w/v) chloramphenicol, and incubated at 28 °C for 5 days. Then, colonies were picked and streaked onto potato dextrose agar (PDA) plates for single-colony isolation. Isolated fungal strains were inoculated into 50 mL of basal medium containing 1.0% (w/v) microcrystalline cellulose and 0.5% (w/v) beechwood xylan in a 500-mL Erlenmeyer flask, and cultivated under shaking at 220 rpm and 28 °C for 5 days. Cells were removed from the culture broth by centrifugation at 15,000×*g* for 10 min at 4 °C, and the supernatant was filtered through a 0.20-μm cellulose acetate membrane filter (13CP020AN; Advantec). About 110 strains of biomass-degrading enzyme-producing fungi were isolated based on their hydrolysis activity toward microcrystalline cellulose at pH 5.0.

### Biomass saccharification

The alkaline-pretreated bagasse was used as the saccharification substrate. Saccharification was performed in 9-mL glass screw-top bottles at a loading of 5% (w/v) dry biomass in 100 mM sodium acetate buffer, pH 5.0, using an enzyme loading of 1.0–2.1 mg protein/g dry weight biomass. The protein concentration was determined using the Bradford protein assay with bovine gamma globulin as the standard. The reaction was performed at 50 °C under shaking at 150 rpm for 72 h. Samples obtained following saccharification were filtered (0.2 μm), and the glucose and xylose concentrations were measured by enzymatic assay using a Multifunction Biosensor BF-7 (Oji, Hyogo, Japan) according to the manufacturer’s protocol. Glucose and xylose yields were calculated as follows:

$$\begin{aligned} {\text{Glucose yield }}\left( \% \right) & = \left({\text{the mass of glucose released}}\right) \\ & \quad \times ({162/180})/({\text{the mass of cellulose in the substrate}}) \\ & \quad \times { 1}00 \hfill \\ \end{aligned}$$

$$\begin{aligned} {\text{Xylose yield }}\left( \% \right)&= \left( {\text{the mass of xylose released}} \right) \\ & \quad \times ({132/150})/({\text{the mass of xylan in the substrate}}) \\ & \quad \times 100 \hfill \\ \end{aligned}$$

Experiments were carried out in triplicate, and data are presented as the mean value ± SD (standard deviation).

### Strain identification by phylogenetic analysis

The genomic DNA of KSM-F532 was extracted by physical disruption and modified Marmur’s method [37]. PCR amplification was conducted by means of puReTaq Ready-To-Go PCR beads (Amersham Biosciences, Piscataway, NJ, USA). The nuclear ribosomal internal transcribed spacer (ITS) region, including ITS1, 5.8S, and ITS2, was sequenced. The sequences were assembled using ChromasPro 1.42 (Technelysium Pty, Ltd., Tewantin QLD, Australia). Multiple alignments were performed using CLUSTAL W [38]; the final alignments were manually adjusted. Ambiguous positions and alignment gaps were excluded from the analysis. The Neighbor-Joining (NJ) tree with the Kimura two-parameter model was constructed using MEGA ver.3.1 [39]. A bootstrap test with 1000 replicates was used to assess the reliability of branches [40]. Positions with gaps and regions of uncertain nucleotide alignment were excluded from phylogenetic analyses.

The methods used for DNA extraction, PCR amplification, DNA sequencing, and molecular phylogenetic analyses were performed by TechnoSuruga Laboratory Co., Ltd. (Shizuoka, Japan).

### SDS-PAGE analysis

SDS-PAGE was carried out with Any kD Mini-PROTEAN TGX Precast Protein Gels (Bio-Rad, Hercules, CA) for 35 min at 200 V. The gel was activated for 5 min and imaged using the ChemiDoc MP imaging system (Bio-Rad). Molecular weight of protein band was estimated using Image Lab software (Bio-Rad). As a molecular mass marker, 5 μL of Precision Plus Protein Unstained Standard (Bio-Rad) was used. Unless otherwise noted, 3 μg of protein was loaded in each well.

### Fractionation of biomass-degrading enzymes produced by strain KSM-F532

The culture supernatant of *Penicillium* sp. KSM-F532 was subjected to size-exclusion column chromatography using a preparative HPLC PLC-561 system (GL Sciences Inc., Tokyo, Japan) equipped with TSKgel G2000SW column (21.5 mm × 300 mm) (Tosoh, Tokyo, Japan). 5 mg of the protein was applied, and protein was eluted with 10 mM sodium acetate buffer (pH 5.0) at a flow rate of 4 mL/min, and 4-mL fractions were collected. The chromatograms were monitored at 280 nm using a UV–Vis detector MU 701 (GL Sciences Inc.). All fractions were analyzed by SDS-PAGE. 8 µL of each fraction was used for SDS-PAGE analysis. All fractions were concentrated using Amicon Ultra-4 3K centrifugal filter units (Merck Millipore) and then used for biomass saccharification analysis.

### Protein identification by mass spectrometry (MS)

8 µg of protein produced by KSM-F532 was separated by SDS-PAGE and stained with Bio-Safe Coomassie stain (Bio-Rad). The separated band was cut out from stained gel, digested with trypsin, and identified by TOF/TOF MS analysis using ultraflex TOF/TOF (Bruker Daltonics, Inc., Billerica, USA) operating in reflector mode and positive polarity. Trypsin digestion, TOF/TOF MS analysis, and de novo sequence analysis were performed by Idea Consultants Inc., Pro Phoenix Division (Osaka, Japan).

### RNA-Seq

Total RNA extraction and synthesis of cDNA were performed using the RNeasy Plant Mini Kit (Qiagen, Crawley, UK) according to the manufacturer’s instructions. Total RNA was employed for the next-generation sequencing libraries using the TruSeq RNA Sample Prep Kit (Illumina, San Diego, CA, USA). Libraries were sequenced using the Illumina MiSeq platform. The obtained sequences were quality-filtered and assembled by CLC Genomics Workbench using the default settings. Then, the obtained contigs were analyzed using the NCBI BLASTx program against the NCBI’s nonredundant protein database.

### Multiple alignments and phylogenetic analysis of xylanase

The amino acid sequences of the PspXyn10 and the orthologs were obtained from NCBI. The alignment was created using ClustalW2 [41] on Genetyx Version 12 software (Genetyx). Phylogenetic trees of the amino acid sequences were created using the NJ method under 1000 times bootstrap replications using Genetyx Version 12 software (Genetyx).

### Construction of the *pspxyn10* expression cassette

The PCR primers are listed in Table 2. These primers introduce restriction sites for vector linearization and vector-specific extensions to facilitate cloning, as required. The amplified fragments were ligated using an In-Fusion HD Cloning Kit (Takara Bio, Shiga, Japan). Plasmids, primer pairs, and templates used for construction of the plasmids are listed in Table 3.

**Table 2:** PCR primers

Primer name	Primer sequence
SwaI *cbh1* F	5′ CTAGAGTATTTAAATGAGCGGCCGAAGACGG 3′
SwaI *cbh1* R	5′ TGCAGGTATTTAAATACCTTATGACAAGAGCT 3′
SwaI pUC F	5′ ATTTAAATACCTGCAGGCATGCAAGCTT 3′
SwaI pUC R	5′ ATTTAAATACTCTAGAGGATCCCCGGGT 3′
*amdS* F	5′ TGCGCACATCATTGGATAGG 3′
*amdS* R	5′ TGCGCACTGGAAACGCAACC 3′
*cbh1*-*amdS* F	5′ CGTTTCCAGTGCGCAGACCGTCACAAGGGACGCAA 3′
*cbh1*-*amdS* R	5′ CCAATGATGTGCGCACTCGGCTACGTTGTCATCGT 3′
P*cbh1* R	5′ GATGCGCAGTCCGCGGTTGA 3′
T*cbh1* F	5′ TAAAGCTCCGTGGCGAAAGCG 3′
P*cbh1*-*pspxyn* F	5′ CGCGGACTGCGCATCATGGTTTGCTTGTCTACCAA 3′
*pspxyn*-T*cbh1* R	5′ CGCCACGGAGCTTTAAAGACATTGCGAGTACCAAG 3′
SwaI *xyn2* F	5′ CTAGAGTATTTAAATTACAAGTCTCCTCTTTC 3′
SwaI *xyn2* R	5′ TGCAGGTATTTAAATTCACGCCTACGCTCCCAA 3′
SwaI *pyr4* F	5′ CTAGAGTATTTAAATGCCTCTTCTTTGTGCTTTTCT 3′
SwaI *pyr4* R	5′ TGCAGGTATTTAAATATATGGAAGCTGATATCGTCG 3′
*xyn2*-*pyr4* F	5′ ACAACGAGAAAAGAAGGACCCTGGGGCAGGTTGCA 3′
*xyn2*-*pyr4* R	5′ ATATCTGGTGGATCTAGGGGGCTCTTCTTTTGTGA 3′
*pyr4* F	5′ TTCTTTTCTCGTTGTCTCACTCT 3′
*pyr4* R	5′ AGATCCACCAGATATGTCAGGA 3′
P*xyn2* R	5′ GTTGATGTCTTCTTGCTTCA 3′
T*xyn2* F	5′ TAAAGGGGGCTCTTCTTTTG 3′
P*xyn2*-*pspxyn F*	5′ CAAGAAGACATCAACATGGTTTGCTTGTCTACCAA 3′
*pspxyn*-T*xyn2* R	5′ GAAGAGCCCCCTTTAAAGACATTGCGAGTACCAAG 3′

**Table 3:** Plasmids, PCR templates, and primers used for plasmid construction

Plasmid	Template	Forward primer	Reverse primer
pUC-*cbh1*	gDNA of PC-3-7	SwaI *cbh1* F	SwaI *cbh1* R
	pUC118	SwaI pUC F	SwaI pUC R
pUC-*cbh1*-*amdS*	pUC-*cbh1*	*cbh1*-*amdS* F	*cbh1*-*amdS* R
	pUC-*amdS*	*amdS* F	*amdS* R
pUC-P*cbh1*-*pspxyn10*-*amdS*	pUC-*cbh1*-*amdS*	T*cbh1* F	P*cbh1* R
	cDNA of KSM-F532	P*cbh1*-*pspxyn* F	*pspxyn*-T*cbh1* R
pUC-*xyn2*	gDNA of PC-3-7	SwaI *xyn2* F	SwaI *xyn2* R
	pUC118	SwaI pUC F	SwaI pUC R
pUC-*xyn2*-*pyr4*	pUC-*xyn2*	*xyn2*-*pyr4* F	*xyn2*-*pyr4* R
	gDNA of PC-3-7	*pyr4* F	*pyr4* R
pUC-P*xyn2*-*pspxyn10*-*pyr4*	pUC-*xyn2*-*pyr4*	T*xyn2* F	P*xyn2* R
	cDNA of KSM-F532	P*xyn2*-*pspxyn* F	*pspxyn*-T*xyn2* R

#### Construction of pUC-P*cbh1*-*pspxyn*10-*amdS*

The promoter, ORF, transcription terminator, and 3′ flanking sequences of *T. reesei cbh1* were amplified from the genomic DNA of *T. reesei* strain PC-3-7 using the primers swaI *cbh1* F and swaI *cbh1* R. A vector fragment was amplified by inverse PCR using pUC118 (Takara Bio) as the template with the primers swaI pUC F and swaI pUC R. The amplified fragments were ligated with an In-Fusion HD Cloning Kit to yield pUC-*cbh1*.

The *amdS* (encoding an *A. nidulans* acetamidase gene) was amplified from pUC-*amdS* using the primers *amdS* F and *amdS* R [42]. The plasmid pUC-*amdS* was kindly provided by Prof. W. Ogasawara. The fragment generated by inverse PCR using pUC-*cbh1* with the primers *cbh1*-*amdS* F and *cbh1*-*amdS* R was fused to the *amdS* fragment to generate pUC-*cbh1*-*amdS*.

A vector fragment was generated by inverse PCR using pUC-*cbh1*-*amdS* with the primers P*cbh1* R and T*cbh1* F. Full-length *pspxyn*10 cDNA was obtained by PCR using a cDNA library of KSM-F532 as the template with the primers P*cbh1*-*pspxyn* F and *pspxyn*-T*cbh1* R. These fragments were ligated to yield pUC-P*cbh1*-*pspxyn*10-*amdS*.

#### Construction of pUC-P*xyn2*-*pspxyn*10-*amdS*

The promoter, ORF, transcription terminator, and 3′ flanking sequences of *T. reesei xyn2* were amplified from the genomic DNA of *T. reesei* strain PC-3-7 using the PCR primers swaI *xyn2* F and swaI *xyn2* R. A vector fragment generated by inverse PCR using pUC118 and the amplified fragments were ligated to yield pUC-*xyn2*.

An *xyn2* vector fragment was amplified by inverse PCR using pUC-*xyn2* with the primers *xyn2*-*pyr4* F and *xyn2*-*pyr4* R, and a *pyr4* fragment was obtained by PCR from the genomic DNA of PC-3-7 using the primers *pyr4* F and *pyr4* R. These fragments were ligated to yield pUC-*xyn2*-*pyr4*.

A *xyn2*-*pyr4* vector fragment was generated by inverse PCR using pUC-*xyn2*-*pyr4* with the primers P*xyn2* R and T*xyn2* F, and full-length *pspxyn*10 cDNA was obtained by PCR using a cDNA library of KSM-F532 as the template with the primers P*xyn2*-*pspxyn* F and *pspxyn*-T*xyn2* R. These fragments were ligated to yield pUC-P*xyn2*-*pspxyn*10-*pyr4*.

### Transformation of *T. reesei*

Plasmids were linearized with *Swa*I prior to transformation. Transformation of *T. reesei* was performed using the protoplast-PEG method [42] with a modification in which 20 mg/mL of Yatalase (Takara Bio) was used as a protoplasting enzyme instead of Novozyme 234 (Novozymes, Bagsværd, Denmark). Transformed protoplasts were plated on the minimal transformation medium [2.0% (w/v) glucose, 18.27% (w/v) sorbitol, 0.5% (w/v) (NH_4_)_2_SO_4_, 0.2% (w/v) CaCl_2_, 0.06% (w/v) MgSO_4_, 0.21% (w/v) CsCl, and 0.1%(w/v) trace element solution in 100 mM KH_2_PO_4_ buffer (pH 5.5)] for *pyr4* marker or minimal transformation medium containing 0.06% (w/v) acetamide instead of (NH_4_)_2_SO_4_ for *amdS* marker. Trace element solution contained 500 mg of FeSO_4_·7H_2_O, 200 mg of CoCl_2_, 160 mg of MnSO_4_·H_2_O, and 140 mg of ZnSO_4_·7H_2_O in 100 mL of distilled water. After 2 weeks of incubation at 28 °C, candidate transformants were streaked twice on selective plates (each minimal transformation medium without sorbitol) for several days at 28 °C to single-colony isolation. Then single colonies were transferred to PDA plates to form conidia for 1 week at 28 °C. One transformant was confirmed by colony PCR using KOD FX Neo (Toyobo, Osaka, Japan) according to the manufacturer’s protocol.

### Enzyme assay

Xylanase activity was measured by assaying the reducing sugars released from xylan. The amount of sugar released was determined according to the 3,5-dinitrosalicylic acid (DNS) method of Miller [43]. Xylanase activity was measured using a final concentration of 1.0% (w/v) beechwood xylan (Megazyme, Bray, Ireland) in 50 mM sodium acetate buffer (pH 5.0) at 50 °C for 5 min. Reactions were performed in a total volume of 0.5 mL and stopped by adding an equal volume of DNS reagent. Then the reaction mixture was boiled for 5 min, and the absorbance was measured at 540 nm. One unit of activity was defined as the amount of enzyme that produced 1 μmol of reducing sugar per minute in xylose equivalents. Experiments were carried out in triplicate, and data are presented as the mean value ± SD.

Enzymatic activity was measured by assaying the *p*-nitrophenol released from 1 mM *p*-nitrophenyl xylobioside (Megazyme) in 50 mM sodium acetate buffer (pH 5.0) at 50 °C for 5 min. Reactions were performed in a total volume of 0.5 mL and stopped by adding an equal volume of 1 M Na_2_CO_3_. The released *p*-nitrophenol was quantified by measuring the absorbance at 420 nm. One unit of activity was defined as the amount of enzyme that produced 1 μmol of *p*-nitrophenol per minute from the substrate.

### Purification of PspXyn10

PspXyn10 was purified from culture filtrate of the *pspxyn*10-expressing transformant, C1PX10. C1PX10 was inoculated into the 50 mL of basal medium containing 1.0% (w/v) microcrystalline cellulose and 0.5% (w/v) beechwood xylan in a 500-mL Erlenmeyer flask, and cultivated with shaking at 220 rpm and 28 °C for 5 days. Cells were removed from the culture broth by centrifugation at 15,000×*g* for 10 min, and the supernatant was filtered through a 0.20-μm cellulose acetate membrane filter (13CP020AN; Advantec). PspXyn10 was purified from the culture filtrate by anion-exchange and size-exclusion chromatography in a HPLC PLC-561 system (GL Sciences Inc.). All purification procedures were performed at 4 °C. First, the culture filtrate was subjected to anion-exchange chromatography using a POROS HQ 20 µm column (4.6 mm × 100 mm) (Applied Biosystems, Foster City, CA) equilibrated with 10 mM Tris–HCl buffer (pH 8.0). After the column was washed with the same buffer, the culture filtrate was applied, and proteins were eluted with a linear gradient of 0–0.8 M NaCl in 10 mL of 10 mM Tris–HCl buffer (pH 8.0), followed by 5 mL of 0.8 M NaCl in the same buffer, at a flow rate of 1 mL/min. The chromatograms were monitored at 280 nm using a UV–VIS detector MU 701 (GL Sciences Inc.). Fractions were collected, and their xylanase activities were assayed. Fractions containing xylanase activity were concentrated, desalted, and buffer-exchanged into 10 mM sodium acetate buffer (pH 5.0) using Amicon Ultra-15 3K centrifugal filter units (Merck Millipore).

The concentrated fraction was subjected to size-exclusion HPLC on a TSKgel G2000SW column (21.5 mm × 300 mm). After the column was washed with 10 mM sodium acetate buffer (pH 5.0), the concentrated fraction was applied, and proteins were eluted at a flow rate of 4 mL/min. All fractions were collected, and their xylanase activities were assayed, and then fractions containing xylanase activity were collected, concentrated, and stored at 4 °C. To confirm that the purified protein was PspXyn10, obtained fractions were subjected to SDS-PAGE.

### Enzymatic properties of PspXyn10

The optimal temperature for hydrolysis of xylan was measured using a final concentration of 1.0% (w/v) beechwood xylan in 50 mM sodium acetate buffer (pH 5.0). The enzyme concentration in the reaction mixture was 1 mg/L. Reaction mixtures were incubated for 5 min at temperatures ranging from 30 to 90 °C. The optimal pH for hydrolysis of xylan was determined by incubating the reaction mixture in the presence of appropriate buffers [50 mM sodium acetate buffer (pH 3.0–5.5) or 50 mM sodium phosphate buffer (pH 6.0–7.0)]. The enzyme concentration in the reaction mixture was 1 mg/L. The reaction mixtures were incubated for 5 min at 50 °C. Thermostability was determined by incubating the enzyme (1 mg/L) in 50 mM sodium acetate buffer (pH 5.0) at temperatures ranging from 30 to 90 °C for 10 min. Then, the residual activity for hydrolysis of xylan was quantified at 50 °C for 5 min.

Kinetic parameters were determined using five concentrations of beechwood xylan (0.5–8 g/L) at 45 °C. The enzyme concentration in the reaction mixture was 1 mg/L. The reaction was stopped by adding DNS reagent at appropriate time intervals (1, 2, 3, 5 and 8 min), and the initial rates of reducing sugar formation were determined. Experiments were conducted in triplicate. Kinetic parameters were obtained by linear regression analysis of the Lineweaver–Burk plot by plotting the reciprocal of the reaction velocity against the corresponding reciprocal of the substrate concentration.

### Limited proteolysis of PspXyn10

Papain (0.5 units/g, Wako Pure Chemical Industries, Ltd., Osaka, Japan) was used for limited proteolysis of PspXyn10 by incubating 1 mL of 0.1 mg/mL PspXyn10 containing 0.3 mg of papain powder in 50 mM sodium phosphate buffer (pH 6.5) at 35 °C for 2 h. The papain-digested PspXyn10 was separated from unreacted PspXyn10 by affinity chromatography using microcrystalline cellulose. This separation step was performed at room temperature. Microcrystalline cellulose (300 mg) was loaded in an empty Micro Bio-Spin chromatography column (Bio-Rad), and the column was washed with 1 mL of 20 mM sodium acetate buffer (pH 5.0) and centrifuged at 1000×*g* for 1 min. The proteolysis reaction mixture (500 μL) was loaded, and the flow-through was collected as a non-cellulose-binding fraction by centrifugation at 1000×*g* for 1 min. The column was washed four times by adding 500 μL of 20 mM sodium acetate buffer (pH 5.0) containing 1 M NaCl and centrifuging at 1000×*g* for 1 min. Then, the cellulose-binding fraction was eluted three times with 0.5 mL of Milli-Q water by centrifugation at 1000×*g* for 1 min. To confirm that the nonbinding and binding fractions were PspXyn10ΔCBM and PspXyn10, respectively, the fractions were subjected to SDS-PAGE, the xylanase activity assay and the cellulose adsorption assay.

### Cellulose adsorption assay

The experiments were carried out in 50 mM sodium acetate buffer (pH 5.0) with or without 5.0% (w/v) microcrystalline cellulose Avicel PH 101 (Sigma-Aldrich, St. Louis, USA) in a 1.0 mL volume. The samples were incubated with 5 μg of protein for 30 min at 50 °C with shaking at 150 rpm. After adsorption, the supernatant was separated by centrifugation at 10,000×*g* for 10 min at room temperature, and subjected to the xylanase activity assay. The adsorption rate was measured by subtracting the xylanase activity in the supernatant from the total activity loaded. Adsorption experiments were carried out in triplicate, and average values are reported.

## Results and discussion

### Saccharification of alkaline-pretreated bagasse by the enzyme preparations

First, we evaluated the saccharification efficiency of alkaline-pretreated bagasse by the enzyme preparations JN11H and JN13H produced by recombinant *T. reesei* X3AB1 and E1AB1, respectively. Saccharification was performed using JN11H or JN13H at pH 5.0 and 50 °C for 72 h, and the enzyme dose was 1.0 or 2.0 mg/g-biomass. When treated with JN11H, the glucose and xylose yields were 43 ± 1 and 46 ± 1% (1.0 mg/g-biomass) and 73 ± 1 and 68 ± 1% (2.0 mg/g-biomass), respectively. When treated with JN13H, the glucose and xylose yields were 43 ± 1 and 49 ± 0% (1.0 mg/g-biomass) and 69 ± 1 and 70 ± 2% (2.0 mg/g-biomass), respectively. It has been reported that JN13H, which contains TrXyn3 in the preparation, is suitable for use with alkaline-pretreated biomass such as sodium hydroxide-pretreated rice straw [29]. TrXyn3 belongs to glycoside hydrolase family 10 (GH10), while *T. reeei* XYN1 (TrXyn1) and XYN2 (TrXyn2), which are contained in the JN11H and JN13H, belong to GH11 [44], and it was reported that GH11 xylanases cleave in unsubstituted regions of the arabinoxylan backbone, whereas GH10 xylanases cleave in decorated regions [45]. Nakazawa et al. have suggested that the enhanced saccharification efficiency of JN13H against alkaline-pretreated rice straw may have resulted from the production of additional xylose from sites cleaved by TrXyn3 (not by TrXyn1 and TrXyn2) and the subsequent exposure of new digestible sites of cellulose caused by xylan removal. However, for the alkaline-pretreated bagasse, the previously reported difference between JN11H and JN13H was not confirmed. This result indicated that the effect of TrXyn3 on saccharification of alkaline-pretreated bagasse is smaller than that of alkaline-pretreated rice straw. Thus, to enhance the saccharification efficiency of alkaline-pretreated bagasse, it seemed necessary to identify an enzyme that has synergistic effects with JN11H enzyme preparation.

### Isolation of *Penicillium* sp. strain KSM-F532

Biomass-degrading enzyme-producing fungi were isolated from a soil sample collected in Tochigi, Japan. These fungal strains were inoculated into basal medium containing cellulose and xylan and cultured. We selected about 110 fungal strains that produced cellulases in culture medium with good reproducibility. The supernatants of these strains containing biomass-degrading enzymes were used for subsequent biomass saccharification.

Biomass saccharification was performed using a mixture of JN11H (1.0 mg/g-biomass) and each culture broth (0.2 mg/g-biomass). It was observed that strain KSM-F532 acted the most synergistically with JN11H: When using only the JN11H, the glucose and xylose yields were 42 ± 3 and 43 ± 1% (1.0 mg/g-biomass) and 46 ± 4 and 46 ± 3% (1.2 mg/g-biomass), respectively; when using the mixture of JN11H (1.0 mg/g-biomass) and the culture broth of strain KSM-F532 (0.2 mg/g-biomass), the glucose and xylose yields increased to 60 ± 2 and 58 ± 1%, respectively.

Strain KSM-F532 was identified as *Penicillium* sp. by phylogenetic analysis using ITS-5.8S rDNA sequencing. The ITS-5.8S rDNA of strain KSM-F532 (Accession No. LC315644) showed the closest match to that of *Penicillium sclerotiorum* JCM 22742^T^ (95.4% identity).

### Fractionation and identification of biomass-degrading enzymes from KSM-F532

**Fig. 1:**
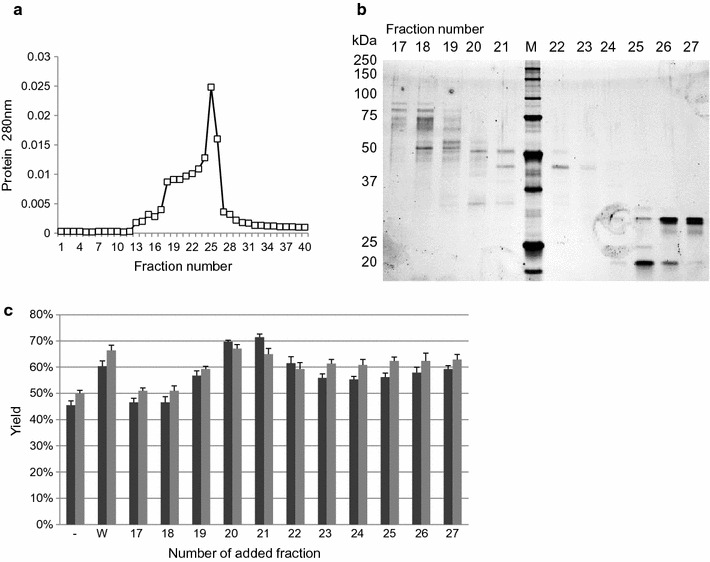
Fractionation of culture filtrate produced by strain KSM-F532 and the saccharification efficiency of the fractions. **a** Elution profile of extracellular proteins from KSM-F532 culture supernatant by gel-filtration chromatography. **b** SDS-PAGE of each fraction; 8 µL of each fraction was loaded (fractions 17–27 shown). Lane M: Precision Plus Protein Unstained Standard (Bio-Rad). **c** Saccharification of alkaline-pretreated bagasse using a mixture of enzyme preparation JN11H (1.0 mg/g-biomass) and fractionated enzymes (0.1 mg/g-biomass) produced by KSM-F532. Saccharification experiments were carried out at pH 5.0, 50 °C with 5% (w/v) alkaline-pretreated bagasse. Black bars indicate glucose yield and gray bars indicate xylose yield. The glucose and xylose yields were calculated from the mass of glucose and xylose released after 72 h. The glucose and xylose yields represent the mean of triplicate experiments. Error bars indicate standard deviations. *–* no fraction was added; *W* whole culture filtrate of KSM-F532 before fractionation was added

The culture filtrate of *Penicillium* sp. strain KSM-F532 was fractionated by gel-filtration chromatography (Fig. 1a), and the fractions obtained were concentrated. SDS-PAGE analysis of each fraction is shown in Fig. 1b. All obtained fractions were used for saccharification of alkaline-pretreated bagasse. Enzymatic hydrolysis was performed using a mixture of JN11H (1.0 mg/g-biomass) and each fraction (0.1 mg/g-biomass). Addition of fractions 20 and 21 resulted in higher saccharification efficiency than JN11H alone (Fig. 1c). A protein of about 55 kDa was common to fractions 20 and 21, and the internal amino acid (AA) sequence of this protein was determined by MS/MS and de novo sequence analysis. Three internal AA sequences were determined, LYYNDYNLESAGAK, NHLTNVVTHYK, and YAWDVVNEGLNDDGTYR, which have high identity to glycoside hydrolase family 10 (GH10) xylanases. Thus, this enzyme was designated PspXyn10.

### Identification of full-length PspXyn10 by cDNA analysis

**Fig. 2:**
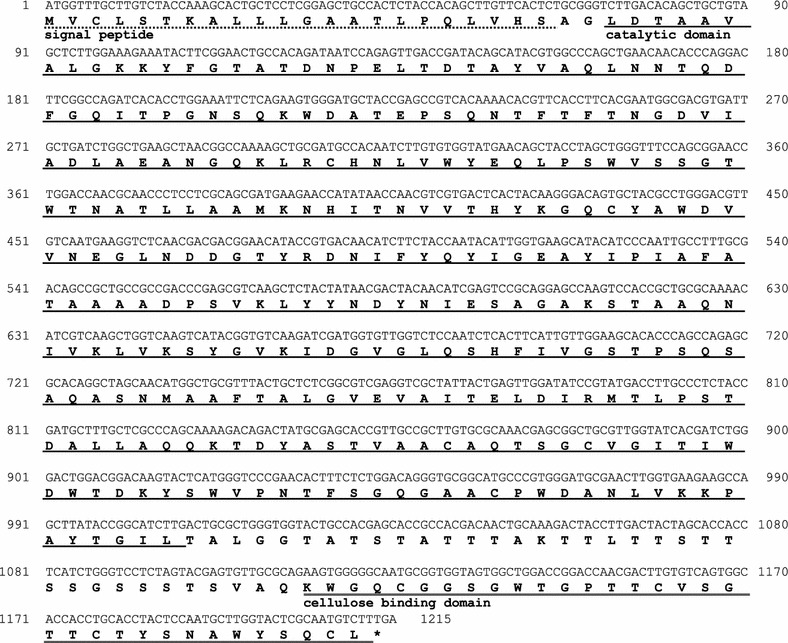
Nucleotide sequence of *pspxyn10* cDNA and deduced amino acid sequence. The predicted signal peptide (dotted line), catalytic domain (solid line), and cellulose-binding domain (CBM1; double line) are indicated

To identify the full-length amino acid sequence of PspXyn10, RNA-Seq analysis was performed. The total RNA of strain KSM-F532 was used for next-generation sequencing, and the complete cDNA sequence of the *pspxyn*10 gene (1215 bp, encoding 404 amino acids) was obtained (Fig. 2). The cDNA sequence was deposited in Gene Bank (Accession No. LC315645).

The SignalP program predicted a putative signal sequence cleavage site between residues Ser-22 and Ala-23. Mature amino acid sequence analysis of PspXyn10 using the NCBI conserved domains search program revealed a modular enzyme composed of two domains (in order from the N-terminus): a catalytic domain (Leu-25 to Leu-336) of GH10 family enzymes and a carbohydrate-binding module family 1 (CBM1) domain (Lys-372 to Leu-404). Comparison of the mature amino acid sequence of PspXyn10 with those of proteins registered in the NCBI database was performed. The xylanase of *Penicillium brasilianum* (OOQ87260) had 84% identity with PspXyn10 and those of *P. subrubescens* (OKO90312), *P. antarcticum* (OQD79351), and *Rasamsonia emersonii* CBS 393.64 (XP_013330999) had 79, 77, and 77% identity, respectively. Multiple sequence alignment of these sequences with TrXyn3 and phylogenetic analysis were performed using Clustal W2 and results are shown in Additional file 1: Figure S1.

This modular organization (GH10 + CBM1) was observed in *Penicillium*, *Talaromyces*, *Aspergillus*, and several other fungal genera, but was not conserved widely. One well-studied xylanase with a CBM1 domain is xyl10A from *Talaromyces cellulolyticus* (GAM37231), and PspXyn10 had 67% identity with this xylanase [46].

The predicted molecular weight of PspXyn10 without the putative signal peptide was 40,521 Da. This size of PspXyn10 was lower than its estimated molecular size of 55 kDa by SDS-PAGE. It was reported that the linker region was highly modified by O-glycosylation in fungal cellulases possessing a linker and a CBM1 domain [47]; we suggest this is also the case for PspXyn10.

### Cloning, expression, and purification of PspXyn10

**Fig. 3:**
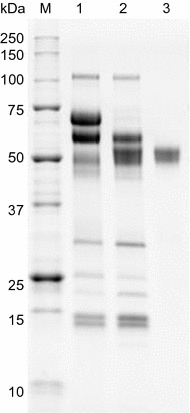
SDS-PAGE of purified PspXyn10. SDS-PAGE was carried out with Any kD Mini-PROTEAN TGX Precast Protein Gels (Bio-Rad, Hercules, CA), and the gel was activated and imaged using the ChemiDoc MP imaging system (Bio-Rad). 3 μg (Lane 1 and 2) or 0.5 μg (Lane 3) of protein were loaded on each gel. Lanes: M, Precision Plus Protein Unstained Standard: (1) enzyme preparation from *T. reesei* strain PC-3-7; (2) enzyme preparation from C1PX10; (3) purified PspXyn10

The *pspxyn*10 gene was cloned from the cDNA of strain KSM-F532. *T. reesei* PC-3-7 was transformed with linearized pUC-P*cbh1*-*pspxyn*10-*amdS* containing the *pspxyn*10 ORF under the control of the *cbh1* promoter. A homologous recombination transformant carrying *pspxyn*10 at the *cbh1* locus was identified by colony PCR analysis. The transformant, designated C1PX10, was cultured in Erlenmeyer flask for 5 days. Analysis of the culture supernatant by SDS-PAGE showed the CBH1 protein band of 60 kDa disappeared, and a new band of about 55 kDa appeared (Fig. 3), in agreement with the molecular mass of KSM-F532 PspXyn10.

The culture filtrate was fractionated by POROS HQ ion-exchange chromatography. The fractions having xylanase activity were concentrated and subsequently fractionated on a TSKgel size-exclusion column. The purified enzyme showed a single band on SDS-PAGE (Fig. 3).

### Enzymatic properties of PspXyn10

**Fig. 4:**
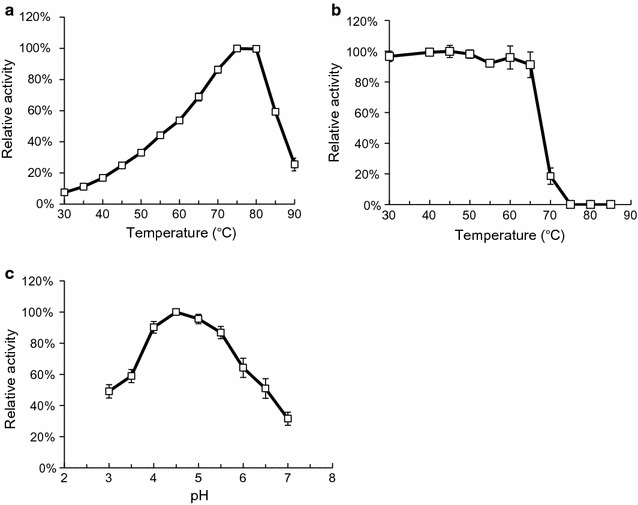
Enzymatic characteristics of purified PspXyn10. **a** Effect of temperature on the xylanase activity of purified PspXyn10. The enzyme reaction was carried out in 50 mM sodium acetate buffer (pH 5.0) for 5 min for 1% (w/v) beechwood xylan with 1 mg/L enzyme at different temperatures. **b** Effect of temperature on the stability of purified PspXyn10. The enzyme (10 mg/L) was incubated in 50 mM sodium acetate buffer (pH 5.0) at different temperatures for 10 min, and then the residual activity for hydrolysis of beechwood xylan was quantified. The enzyme reaction was carried out in 50 mM sodium acetate buffer (pH 5.0) for 1% (w/v) beechwood xylan with 1 mg/L enzyme at 50 °C for 5 min. **c** Effect of pH on the xylanase activity of purified PspXyn10. The enzyme reaction was carried out in 50 mM sodium acetate buffer (pH 3.0–5.5) or 50 mM sodium phosphate buffer (pH 6.0–7.0) for 5 min for 1% (w/v) beechwood xylan with 1 mg/L enzyme at 50 °C. The values of relative activities are the means of three replicate. Error bars indicate standard deviations

The effect of temperature on the activity of purified PspXyn10 was determined. The optimal temperature for PspXyn10 activity was 75 °C (Fig. 4a). Recent studies have documented that thermostable enzymes are suitable for lignocellulose saccharification because their enhanced stability leads to retention of activity over longer times at both moderate and elevated temperatures and decreases the amount of enzyme needed for saccharification [48, 49]. However, almost all fungal xylanases previously reported were mesophilic, and very few thermophilic fungal xylanases were reported. The *Bispora* sp. xylanase, Xyn10C, showed an optimal activity at 80 °C, which is higher than any other fungal xylanases [48, 50]. PspXyn10 showed an optimal temperature close to that of Xyn10C, indicating that PspXyn10 is suitable for high-temperature saccharification. The thermostability of PspXyn10 was determined by incubating the enzyme at 20–90 °C for 10 min (Fig. 4b). More than 80% of the xylanase activity was maintained at 65 °C.

The effect of pH on the activity of the PspXyn10 was also determined (Fig. 4c). The optimal pH of PspXyn10 was 4.5, and the xylanase activity between pH 3.0 and pH 6.5 was more than 50% of that at pH 4.5. Since the enzyme preparations from *T. reesei* are generally used around pH 5.0, it was indicated that PspXyn10 is suitable for being used together with them.

The kinetic parameters *K*_m_ and *V*_max_ of PspXyn10 were determined from Lineweaver–Burk (double-reciprocal) plots of xylanase activity at 45 °C using various concentrations of beechwood xylan as the substrate (Table 4). The *K*_m_, *V*_max_, *k*_cat_, and *k*_cat_/*K*_m_ of the xylanase were 2.2 ± 0.3 mg/mL, 332 ± 29 μmol/min/mg, 221 ± 20/s, and 102 ± 4 mL/s/mg, respectively. It was reported that the *K*_m_ and *V*_max_ of TrXyn3 against birchwood xylan are 2.02 mg/mL and 256 μmol/min/mg, respectively [51], and *K*_m_, *k*_cat_, and the *k*_cat_/*K*_m_ of xyl10A from *Talaromyces cellulolyticus* against birchwood xylan are 2.51 mg/mL, 232/s, and 92.2 mL/s/mg, respectively [46]. Compared with these GH10 xylanases, it seemed that the kinetic parameters of PspXyn10 did not differ much from those of general GH10 xylanases.

**Table 4:** Kinetic parameters for hydrolysis of beechwood xylan by PspXyn10

*V*_max_	50 ± 4	mg/min/mg
	332 ± 29	μmol/min/mg
*K*_m_	2.2 ± 0.3	mg/mL
	14.6 ± 1.8	mM
*k*_cat_	221 ± 20	/s
*k*_cat_/*K*_m_	102 ± 4	/s/mg/mL

### Synergistic saccharification using JN11H and purified PspXyn10

**Fig. 5:**
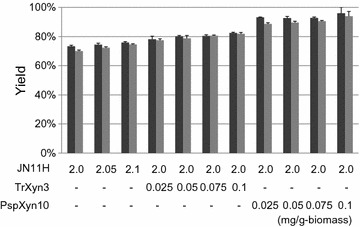
Saccharification of alkaline-pretreated bagasse using a mixture of JN11H (2.0 mg/g-biomass) and TrXyn3 (0.025-0.1 mg/g-biomass) or JN11H (2.0 mg/g-biomass) and purified PspXyn10 (0.025–0.1 mg/g-biomass). Saccharification experiments were carried out at pH 5.0, 50 °C with 5% (w/v) alkaline-pretreated bagasse. Enzyme dosage is indicated at the bottom of each bar. Black bars indicate glucose yield and gray bars indicate xylose yield. The glucose and xylose yields were calculated from the mass of glucose and xylose released after 72 h. The glucose and xylose yields represent the means of triplicate experiments. Error bars indicate standard deviations

**Fig. 6:**
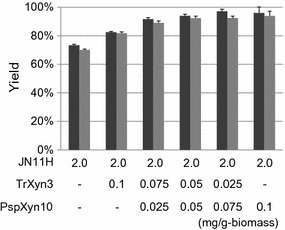
Saccharification of alkaline-pretreated bagasse using a mixture of JN11H, TrXyn3, and purified PspXyn10. 0.1 mg/g-biomass of a mixture of PspXyn10 and TrXyn3, and 2 mg/g-biomass of JN11H were used for saccharification. Saccharification experiments were carried out at pH 5.0, 50 °C with 5% (w/v) alkaline-pretreated bagasse. Enzyme dosage is indicated at the bottom of each bar. Black bars indicate glucose yield and gray bars indicate xylose yield. The glucose and xylose yields were calculated from the mass of glucose and xylose released after 72 h. The glucose and xylose yields represent the means of triplicate experiments. Error bars indicate standard deviations

To confirm the synergy between JN11H and PspXyn10, saccharification of alkaline-pretreated bagasse was performed using an enzyme mixture of JN11H (2.0 mg/g-biomass) and purified PspXyn10 (0.025–0.1 mg/g-biomass) or JN11H (2.0 mg/g-biomass) and TrXyn3 (0.025–0.1 mg/g-biomass) (Fig. 5). When using only the JN11H enzyme preparation, the glucose and xylose yields were 72 ± 1 and 69 ± 1% (2.0 mg/g-biomass) and 74 ± 1 and 73 ± 1% (2.1 mg/g-biomass), respectively. When using a mixture of JN11H (2.0 mg/g-biomass) and PspXyn10 (0.1 mg/g-biomass), the glucose and xylose yields were 97 ± 4 and 95 ± 3%, respectively; when using a mixture of JN11H (2.0 mg/g-biomass) and TrXyn3 (0.1 mg/g-biomass), the glucose and xylose yields were 80 ± 1 and 80 ± 1%, respectively. Therefore, a highly synergistic effect was observed between JN11H and PspXyn10, and it was estimated that PspXyn10 was the main contributor to the synergistic effect observed to the total biomass-degrading enzymes produced by strain KSM-F532. In contrast, the improvement in the saccharification efficiency by adding TrXyn3 was less than half that of adding PspXyn10. Moreover, to confirm the synergistic effect between PspXyn10 and TrXyn3, saccharification of alkaline-pretreated bagasse was performed using an enzyme mixture of JN11H, purified PspXyn10 and TrXyn3 (Fig. 6). A total of 0.1 mg/g-biomass of a mixture of PspXyn10 and TrXyn3 and 2.0 mg/g-biomass of JN11H were used for saccharification. As shown in Fig. 6, the glucose and xylose yields increased as the content of PspXyn10 increased. This result indicated that synergistic effect was not present between PspXyn10 and TrXyn3 and PspXYN10 promotes saccharification of alkaline-pretreated bagasse by acting at site where TrXYN3 is not active. PspXyn10 and TrXyn3 differ in that the former has a CBM1 domain, and the latter does not, therefore, investigation of the importance of the CBM1 domain in saccharification was undertaken.

### Evaluation of the importance of the CBM1 domain in saccharification

**Fig. 7:**
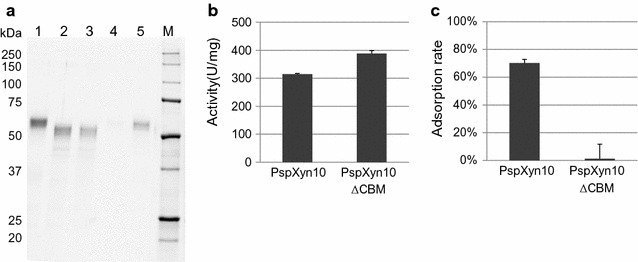
Characterization of PspXyn10ΔCBM. **a** SDS-PAGE of cellulose-binding and nonbinding fractions, fractionated from limited (papain) degraded PspXyn10. Lanes: 1, purified PspXyn10; 2, nonbinding fraction 1; 3, nonbinding fraction 2; 4, binding fraction 1; 5, binding fraction 2; M, Precision Plus Protein Unstained Standard. **b** Specific xylanase activities of PspXyn10 and PspXyn10ΔCBM. The enzyme reaction was carried out in 50 mM sodium acetate buffer (pH 5.0) for 5 min for 1% (w/v) beechwood xylan with 1 mg/L enzyme at 50 °C. The values of specific activity are the means of three replicates. Error bars indicate standard deviations. **c** Adsorption rates of PspXyn10 and PspXyn10ΔCBM to microcrystalline cellulose. 5 μg of protein were incubated with or without 5.0% (w/v) microcrystalline cellulose Avicel PH 101 in 50 mM sodium acetate buffer (pH 5.0) for 30 min at 50 °C under shaking at 150 rpm. Then the supernatant was separated by centrifugation, and subjected to xylanase activity assay. The adsorption rate was measured by subtracting the xylanase activity in supernatant from the total activity loaded. The values of adsorption rate are the means of three replicates. Error bars indicate standard deviations

It was reported that the CBH1 from *T. reesei* was degraded to a catalytic domain and a cellulose-binding-domain by limited proteolysis using papain [52]. To obtain PspXyn10ΔCBM, from which the CBM1 domain was removed, limited proteolysis of PspXyn10 was carried out. PspXyn10 and papain were mixed and reacted, and then the cellulose-binding fraction and the nonbinding fraction were separated by affinity chromatography using a cellulose column. The non-cellulose-binding fraction was recovered from the flow-through, and the cellulose-binding fraction was eluted with Milli-Q water, then these fractions were concentrated. The non-cellulose-binding fraction showed a single band of 50 kDa on SDS-PAGE (Fig. 7a). The cellulose-binding fraction only contained a protein of 55 kDa, the same size as PspXyn10. From this result, we suggested that the nonbinding fraction mainly contained PspXyn10ΔCBM, and the cellulose-binding fraction was PspXyn10. The specific activities toward beechwood xylan of the binding fraction and the nonbinding fraction were 315 ± 5 and 388 ± 17 U/mg-protein, respectively (Fig. 7b), and this result indicated that PspXyn10ΔCBM has higher specific activity against soluble xylan than PspXyn10. Moreover, PspXyn10 and PspXyn10ΔCBM were subjected to cellulose adsorption assay. As a result, it was shown that although about 70% of PspXyn10 binds to crystalline cellulose in this condition, PspXyn10ΔCBM hardly bind to the cellulose (Fig. 7c). From these results, it was indicated that PspXyn10ΔCBM maintains its xylanase activity but loses its cellulose-binding activity.

To evaluate the importance of the CBM1 domain, saccharification of alkaline-pretreated bagasse was performed using a mixture of JN11H and PspXyn10 or PspXyn10ΔCBM. When using a mixture of JN11H (2.0 mg/g-biomass) and PspXyn10 (0.1 mg/g-biomass), the glucose yield was 92 ± 2%. On the contrary, when using a mixture of JN11H (2.0 mg/g-biomass) and PspXyn10ΔCBM (0.1 mg/g-biomass), the glucose yield was 84 ± 1%. This result suggested that loss of the CBM1 domain resulted in decreased saccharification efficiency of PspXyn10 against alkaline-pretreated bagasse to about the same level as that of TrXyn3 (Fig. 5), and that the cellulose-binding activity of xylanase PspXyn10 was one of the reasons for the high saccharification activity shown by this enzyme.

Based on the results in this study, it became clear that PspXyn10 promotes the saccharification of alkaline-pretreated bagasse, and while PspXyn10ΔCBM shows increased activity against soluble xylan compared with PspXyn10, its ability to adsorb to cellulose is eliminated, and its improvement of the saccharification efficiency of alkaline-pretreated bagasse was decreased. These results indicate that adsorption to cellulose by CBM1 is important for PspXyn10 to enhance the saccharification of alkaline-pretreated bagasse. Many studies have reported the importance of the CBM1 of fungal cellulases. It has been reported that the removal of the CBM1 causes the decrease of cellulase activity against insoluble substrates, but not on soluble substrate [53–55]. Moreover, it has been suggested that CBM1 of CBH assists the processive hydrolysis of cellulose [56]. However, only Inoue et al. have reported the importance of the CBM1 of fungal xylanase on biomass saccharification [46]. Inoue et al. suggested that the function of the CBM1 of fungal xylanase is to increase the effective enzyme concentrations on the insoluble polysaccharide surfaces to target the catalytic domain to the substrate. In our study, when TrXyn3 was added to JN11H, the glucose yield was saturated at about 80%, but when a small amount of PspXyn10 was added to JN11H, the glucose yield increased to over 90% (Fig. 5). Moreover, no synergistic effect was observed between TrXyn3 and PspXyn10 (Fig. 6). From these results, we consider that the alkaline-pretreated bagasse may contain some kind of xylan which inhibits the cellulose degradation by cellulases, and this xylan is difficult to be degraded by TrXyn3 but is degradable by PspXyn10. It is considered that xylan may either be closely associated with cellulose or loosely form a three-dimensional structure in the lignocellulosic biomass [56], Moreover, it was reported that only the loosely bound fraction of xylan could be degraded by xylanase and the xylan tightly associated to cellulose still remained after hydrolysis by the enzymes [57]. Also, it has been reported that the tightly adsorbed xylan inhibits degradation of the cellulose by cellulase [57–59]. Based on these previous studies and our study, we consider that PspXyn10 may promote saccharification efficiency by removing the xylan associating with cellulose tightly, and to remove this xylan efficiently, adsorption to cellulose surface by CBM1 is important. Therefore, more studies are needed to understand the detailed xylan structures of various substrates and how PspXyn10 enhances the saccharification efficiency.

### Construction of the PspXyn10-expressing strain and its saccharification efficiency

For economic sugar and bioethanol production, it is desirable to produce an enzyme mixture by only culturing one strain. Therefore, construction of a strain that can produce an enzyme mixture suitable for saccharification of alkaline-pretreated bagasse was performed.

*Trichoderma reesei* strain X3AB1Δ*pyr4* was transformed with the expression cassette P*xyn2*-*pspxyn*10 containing a *pspxyn*10 cDNA under the control of the *xyn2* promoter, which is a xylan-inducible high-expression promoter. The transformant, designated X2PX10, was cultured in a 250-mL jar fermenter for 4 days, and the filtered supernatant was used as enzyme preparation JNK25H.

A distinct band for PspXyn10 was not obtained from SDS-PAGE analysis of JNK25H because the band size of *T. reesei* EG2 is the same as that of PspXyn10 (Additional file 2: Figure S2). To estimate the content of PspXyn10 in the JNK25 preparation, the enzymatic activity of purified PspXyn10, JN11H, and JNK25H against *p*-nitrophenyl xylobioside (pNPX2) was assayed. The specific activities of PspXyn10, JN11H, and JNK25H against pNPX2 were 361 ± 7, 0.8 ± 0.1 and 18 ± 1 U/mg, respectively, and from the difference of the specific activity between JN11H and JNK25H, it was estimated that about 0.05 mg of PspXyn10 was present in 1 mg of JNK25H preparation.

Saccharification of alkaline-pretreated bagasse was performed using 1.0 or 2.0 mg/g-biomass of JNK25H. The glucose and xylose yields were 70 ± 3 and 60 ± 1% (1.0 mg/g-biomass) and 90 ± 3 and 81 ± 3% (2.0 mg/g-biomass), respectively. The glucose and xylose yields using each enzyme preparation are summarized in Table 5. Compared with JN11H and JN13H, JNK25H showed high glucose and xylose yields, and, when using JNK25H, the enzyme dosage could be reduced to about half of that for JN11H or JN13H. From this result, we concluded that the enzyme JNK25H is suitable for saccharification of alkaline-pretreated bagasse and when using the strain X2PX10, sugar and bioethanol production from sugarcane bagasse can be carried out at low cost.

**Table 5:** Glucose and xylose yields of alkaline-pretreated bagasse using enzyme preparations

Preparation	Yields			
	1 mg/g-biomass		2 mg/g-biomass	
	Glucose (%)	Xylose (%)	Glucose (%)	Xylose (%)
JN11H	43 ± 1	46 ± 1	73 ± 1	68 ± 1
JN13H	43 ± 1	49 ± 0	69 ± 1	70 ± 2
JNK25H	70 ± 3	60 ± 1	90 ± 3	81 ± 3

## Conclusions

In this study, we initially evaluated the saccharification efficiency of alkaline-pretreated bagasse using two enzyme preparations, JN11H and JN13H. We determined that the same degree of degradation efficiency was observed for both, even though JN13H contains TrXyn3, which was considered effective for alkaline-pretreated biomass decomposition. Therefore, screening for an enzyme to promote saccharification of alkaline-pretreated bagasse was performed, and PspXyn10 was identified from *Penicillium* sp. KSM-F532. Interestingly, both PspXyn10 and TrXyn3 are GH10 family xylanases.

PspXyn10 is modular enzyme and contains a CBM1 domain. PspXyn10ΔCBM was prepared by limited proteolysis, and its synergistic hydrolysis of biomass was evaluated. Removal of the CBM resulted in a decrease in the synergistic effect, although the specific xylanase activity was improved. From this result, we conclude that the CBM1 domain of PspXyn10 contributes to the enhancement of the saccharification efficiency.

Subsequently, we developed the enzyme preparation JNK25H, which contains AaBGL1 and PspXyn10, produced by recombinant *T. reesei* strain X2PX10. This enzyme preparation showed very high saccharification efficiency toward alkaline-pretreated bagasse.

## Additional files

**Additional file 1: Figure S1.** Multiple alignments and phygogenetic tree of PspXyn10, PspXyn10 orthologs and TrXyn3. (a) Multiple alignments of the PspXyn10, PspXyn10 orthologs and TrXyn3. The amino acid sequence of PspXyn10 was compared with those of endo-1,4-beta-xylanase from *Penicillium brasilianum* (OOQ87260), endo-1,4-beta-xylanase D from *Penicillium subrubescens* (OKO90312), endo-1,4-beta-xylanase from *Rasamsonia emersonii* CBS 393.64 (XP_013330999), hypothetical protein PENANT_c053G02808 from *Penicillium antarcticum* (OQD79351), endo-1,4-beta-xylanase Xyl10A from *Talaromyces cellulolyticus* (GAM37231) and TrXyn3 from *Trichoderma reesei* (BAA89465) by multiple alignment. The alignment was created using ClustalW2 on Genetyx Version 12 software (Genetyx). Amino acid numbers are shown on the left and right. *Black boxes* indicate invariant residues. *Gray boxes* indicate the residues conserved in more than half of aligned sequences. (b) Phylogenetic tree of the PspXyn10, PspXyn10 orthologs and TrXyn3. Phylogenetic tree of the amino acid sequences were created using the NJ method under 1000 times bootstrap conditions using Genetyx Version 12 software (Genetyx).

**Additional file 2: Figure S2.** SDS-PAGE of enzyme preparation JN11H and JNK25H. SDS-PAGE was carried out with Any kD Mini-PROTEAN TGX Precast Protein Gels (Bio-Rad, Hercules, CA) and the gel was activated and imaged using the ChemiDoc MP imaging system (Bio-Rad).3 μg of protein were loaded on each gel. Lanes: M, Precision Plus Protein Unstained Standard; 1, JN11H enzyme preparation produced by *T. reesei* strain X3AB1; 2, JNK25H enzyme preparation produced by strain X2PX10. Cellobiohydrolases (CBH 1, CBH2), endoglucanases (EG1 and EG2), xylanases (XYN1 and XYN2) and β-xylosidases (BXL) from *Trichoderma reesei*, and heterologously expressed proteins (β-glucosidase AaBGL1 from *Aspergillus aculeatus* and xylanase PspXyn10 from *Penicillium* sp. KSM-F532) are shown.

## Abbreviations

GH10: glycoside hydrolase family 10
CBM: carbohydrate-binding module
CBH1: cellobiohydrolase 1
EG1: endoglucanase 1
TrXyn3: *Trichoderma reesei* xylanase 3
ORF: open reading frame
PCR: polymerase chain reaction
