# Supplementary material for: A novel GH10 xylanase from Penicillium sp. accelerates saccharification of alkaline-pretreated bagasse by an enzyme from recombinant Trichoderma reesei expressing Aspergillus β-glucosidase
Source: Biotechnol Biofuels. 2017 Nov 21;10:278. doi: 10.1186/s13068-017-0970-2 (PMC5698967; doi:10.1186/s13068-017-0970-2)
Supplement: Supplementary file 1 — Additional file 1: Figure S1. Multiple alignments and phygogenetic tree of PspXyn10, PspXyn10 orthologs and TrXyn3. (a) Multiple alignments of the PspXyn10, PspXyn10 orthologs and TrXyn3. The amino acid sequence of PspXyn10 was compared with those of endo-1,4-beta-xylanase from Penicillium brasilianum (OOQ87260), endo-1,4-beta-xylanase D from Penicillium subrubescens (OKO90312), endo-1,4-beta-xylanase from Rasamsonia emersonii CBS 393.64 (XP_013330999), hypothetical protein PENANT_c053G02808 from Penicillium antarcticum (OQD79351), endo-1,4-beta-xylanase Xyl10A from Talaromyces cellulolyticus (GAM37231) and TrXyn3 from Trichoderma reesei (BAA89465) by multiple alignment. The alignment was created using ClustalW2 on Genetyx Version 12 software (Genetyx). Amino acid numbers are shown on the left and right. Black boxes indicate invariant residues. Gray boxes indicate the residues conserved in more than half of aligned sequences. (b) Phylogenetic tree of the PspXyn10, PspXyn10 orthologs and TrXyn3. Phylogenetic tree of the amino acid sequences were created using the NJ method under 1000 times bootstrap conditions using Genetyx Version 12 software (Genetyx). [file 13068_2017_970_MOESM1_ESM.pdf]

(a)

```
Penicillium sp. KSM-F532     1 ------------------------------------------------AGLDTAAVALGKKYFGTATDNPELTDTAYVAQLNNTQDFGQITPGNSQKWDATEPSQNTFTFTNGDVIADLA 72
Penicillium antarcticum      1 --------------------------MVHLSAIALALAG-VLPQLTQAAGLNTAAVAKGQLYFGSATDNPELSDSAYLTQLSNTDDFGQITPGNSQKWDATEPTQNKFSFAQGDVIADLA 93
Penicillium brasilianum      1 --------------------------MVHLSASSLLLAAGILSKLALGAGLDTAAVAIGKLYFGTATDNPELTDTAYVTQLNNTNDFGQITPGNSQKWDATEPSQNTFTFTNGDTVAKLA 94
Penicillium subrubescens     1 --------------------------MVHLSASSLLLAAGILPTLAYGAGLNTAAVALGKLYFGSATDNGELSDSAYVTQLSNTQDFGQITPGNSQKWDATEPSQNVFTYTNGDTIAKLA 94
Rasamsonia emersonii         1 --------------------------MVRLSPVLLASIAGSGLPLAQAAGLNTAAKAIGLKYFGTATDNPELSDTAYETQLNNTQDFGQLTPANSMKWDATEPEQNVFTFSAGDQIANLA 94
Talaromyces cellulolyticus   1 ------------------------------------------MGHVAQAQLNTAAKAAGLLYFGTAVDNPDLSDSKYIANL-ETADFGQITPANAMKWQPTEPSQGSYTFTQGDQIASLA 77
Trichoderma reesei           1 MKANVILCLLAPLVAALPTETIHLDPELAALRANLTERTADLWDRQASQSIDQLIKRKGKLYFGTATDRGLLQRE--KNAAIIQADLGQVTPENSMKWQSLENNQGQLNWGDADYLVNFA 118

Penicillium sp. KSM-F532    73 EANGQKLRCHNLVWYEQLPSWVSSGTWTNATLLAAMKNHITNVVTHYKGQCYAWDVVNEGLNDDGTYRDNIFYQYIGEAYIPIAFATAAAADPSVKLYYNDYNIESAGA-KSTAAQNIVK 191
Penicillium antarcticum     94 ATNGQKLRCHNLVWHSQLPSWVSSGTWTNATLIAAMKNHITNVVTHYKGKCYAWDVVNEALNEDGSYRSSIFYTTIGEAFLPIAFAAAAAADPSVKLYYNDYNIESAGS-KSTGAQRIVK 212
Penicillium brasilianum     95 EANGQKLRCHNLVWHSQLPSWVTSGTWTNATLVAALKNHITNVVTHYKGQCYAWDVVNEALNEDGTYRTSLWYQTIGEAYIPIAFATAAAADPSVKLYYNDYNIEYSGS-KATGALKIVK 213
Penicillium subrubescens    95 KTNGQKLRCHNLVWHNQLPNWVTSGSWTNATLIAALKNHITNVVTHYKGQCYAWDVVNEALNEDGTYRTSLWYNTIGEAYLPIAFAAAAAADPSVKLYYNDYNIEYSGA-KATGALRIVK 213
Rasamsonia emersonii        95 KANGQMLRCHNLVWYNQLPSWVTSGSWTNETLLAAMKNHITNVVTHYKGQCYAWDVVNEALNDDGTYRSNVFYQYIGEAYIPIAFATAAAADPNAKLYYNDYNIEYPGA-KATAAQNLVK 213
Talaromyces cellulolyticus  78 KSNNDYLRCHNLVWYNQLPSYITSGSWTNATLIAALKEHINGVVTHYKGQCYAWDVVNEALNEDGTYRQNVFYQYIGEAYIPIAFAAAAAADPNAKLYYNDYNIEYAGS-KATGAQRIVK 196
Trichoderma reesei         119 QQNGKSIRGHTLIWHSQLPAWVNN-INNADTLRQVIRTHVSTVVGRYKGKIRAWDVVNEIFNEDGTLRSSVFSRLLGEEFVSIAFRAARDADPSARLYINDYNLDRANYGKVNGLKTYVS 237

Penicillium sp. KSM-F532   192 LVKSYGVKIDGVGLQSHFIVGSTPSQSAQASNMAAFTALGVEVAITELDIRMTLPSTDALLAQQKTDYASTVAACAQTSGCVGITIWDWTDKYSWVPNTFSGQGAACPWDANLVKKPAYT 311
Penicillium antarcticum    213 LIQSYGVKIDGVGLQAHFIVGSTPSLSAQTTNLAAFTALGVEVAYTELDIRMTLPSTSALLAQQSTDYQNTVAACVANVKCVGVTIWDYTDKYSWVPGTFSGQGAACPWDDRLVKKPAYT 332
Penicillium brasilianum    214 LIKSYGVKIDGVGLQAHFIVGSTPSRSNQASTMASYTALGVEVAITELDIRMTLPATDALLAQQSTDYSNTVGACVDTSGCVGVTIWDWTDKYSWVPSTFSGQGAACPWDENYAKKPAYT 333
Penicillium subrubescens   214 LVQSYGVKIDGVGLQAHFIVGSTPSRSNQASTMAAYTALGVEVAITELDIRMTLPSTAALLAQQSTDYASSVGACVDTKGCVGVTIWDWTDKYSWVPGTFSGQGAACPWDSNFVKKPAYT 333
Rasamsonia emersonii       214 LVQSYGARIDGVGLQSHFIVGETPSTSSQQQNMAAFTALGVEVAITELDIRMQLPETEALLTQQATDYQSTVQACANTKGCVGITVWDWTDKYSWVPSTFSGYGDACPWDANYQKKPAYE 333
Talaromyces cellulolyticus 197 LIQAAGGRIDGVGLQSHFIVGQTPSLATQKANMAAFTALGVDVAITELDIRMTLPDTSALQTQQSTDYQTTTTACVQTKGCVGITLWDYTDKYSWVPGTFSGQGDACPWDSNYNKKPAYY 316
Trichoderma reesei         238 KWISQGVPIDGIGSQSHLSGGGGSGTLGALQQLATVPVT--ELAITELDIQG----------APTTDYTQVVQACLSVSKCVGITVWGISDKDSWRASTN-----PLLFDANFNPKPAYN 340

Penicillium sp. KSM-F532   312 GILTALG-GTATST---ATTTAKTTLTTSTTSSG------SSSTSVAQKWGQCGGSGWTGPTTCVSGTTCTYSNAWYSQCL 382
Penicillium antarcticum    333 GILNALG-GSASSTTTAATTLATTTKATTTTTAS------SGGSTGVAHWGQCGGSGWTGGTVCVSGYTCTYTNDWYSQCL 406
Penicillium brasilianum    334 GILTALG-GTATAT---STTTSTTSKSTTTTTTS----STSTSTAVAAHWGQCGGSGWTGPTVCASGYTCTYSNAWYSQCL 406
Penicillium subrubescens   334 GILSALG-GSATST---ATTTLVTSVSSTSTTTSTATTSTSTSTGVAQHWGQCGGSGWTGPTTCASPYTCTYSNAWYSQCL 410
Rasamsonia emersonii       334 GILTGLG-QTVTSTT--YIISPTTSVGTGTTTSSG---GSGGTTGVAQHWEQCGGLGWTGPTVCASGYTCTVINEYYSQCL 408
Talaromyces cellulolyticus 317 GILAGLQSGTGSSSSTSSTTLTTTTTPTTASSTTS--TTSTSATSGAAHWGQCGGIGWSGPTICVSPYTCQVLNPYYSQCL 395
Trichoderma reesei         341 SIVGILQ-------------------------------------------------------------------------- 347
```

(b)

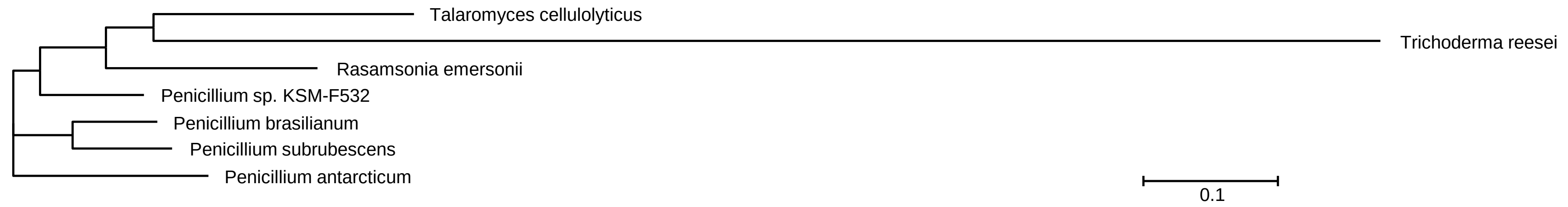

Figure S1 N. Shibata *et. al.*
