# Supplementary figures and images for: A novel GH10 xylanase from Penicillium sp. accelerates saccharification of alkaline-pretreated bagasse by an enzyme from recombinant Trichoderma reesei expressing Aspergillus β-glucosidase
Source: Biotechnol Biofuels. 2017 Nov 21;10:278. doi: 10.1186/s13068-017-0970-2 (PMC5698967; doi:10.1186/s13068-017-0970-2)

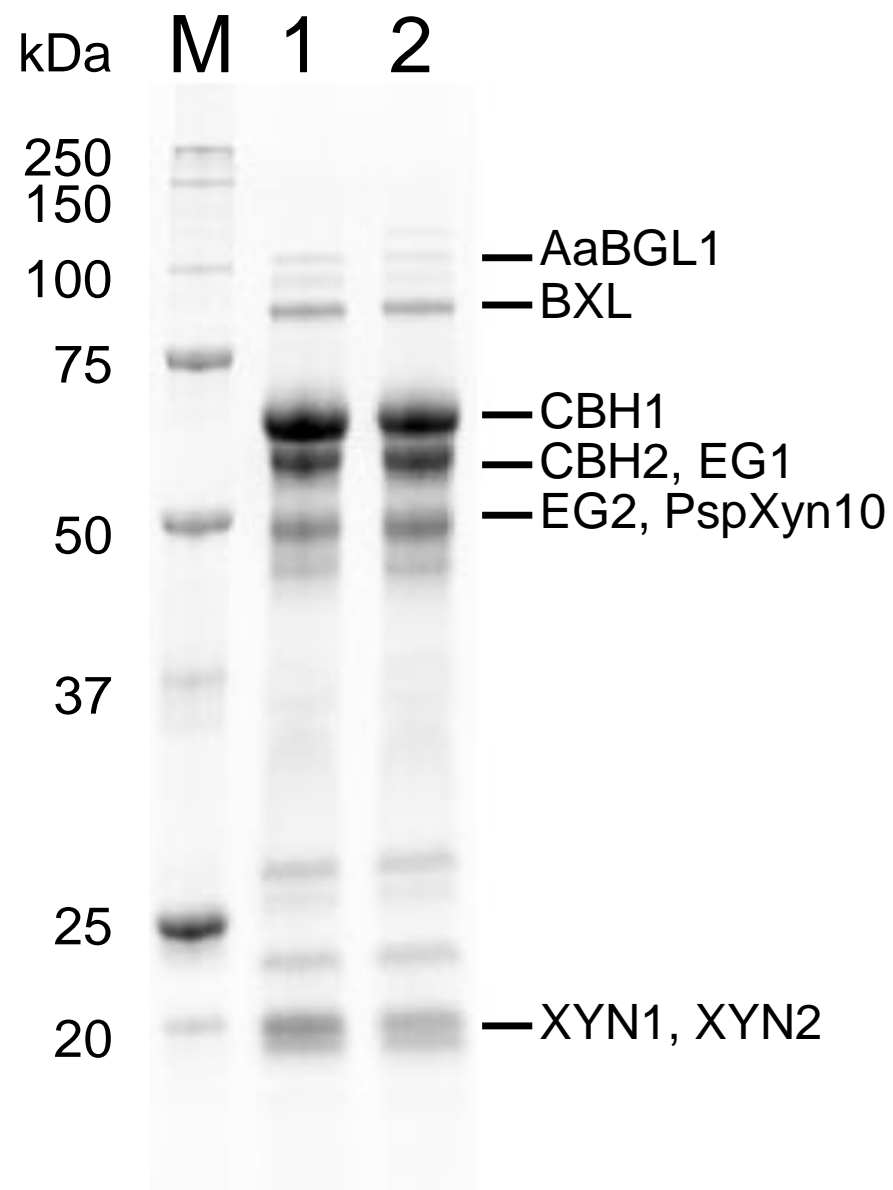

Figure S2 N. Shibata *et. al.*

Supplement: Supplementary file 2 — Additional file 2: Figure S2. SDS-PAGE of enzyme preparation JN11H and JNK25H. SDS-PAGE was carried out with Any kD Mini-PROTEAN TGX Precast Protein Gels (Bio-Rad, Hercules, CA) and the gel was activated and imaged using the ChemiDoc MP imaging system (Bio-Rad).3 μg of protein were loaded on each gel. Lanes: M, Precision Plus Protein Unstained Standard; 1, JN11H enzyme preparation produced by T. reesei strain X3AB1; 2, JNK25H enzyme preparation produced by strain X2PX10. Cellobiohydrolases (CBH 1, CBH2), endoglucanases (EG1 and EG2), xylanases (XYN1 and XYN2) and β-xylosidases (BXL) from Trichoderma reesei, and heterologously expressed proteins (β-glucosidase AaBGL1 from Aspergillus aculeatus and xylanase PspXyn10 from Penicillium sp. KSM-F532) are shown. [file 13068_2017_970_MOESM2_ESM.pdf]
